# Origin, genomic diversity and microevolution of the *Clostridium difficile* B1/NAP1/RT027/ST01 strain in Costa Rica, Chile, Honduras and Mexico

**DOI:** 10.1099/mgen.0.000355

**Published:** 2020-03-16

**Authors:** Enzo Guerrero-Araya, Claudio Meneses, Eduardo Castro-Nallar, Ana M. Guzmán D., Manuel Álvarez-Lobos, Carlos Quesada-Gómez, Daniel Paredes-Sabja, César Rodríguez

**Affiliations:** ^1^​ Millennium Nucleus in the Biology of Intestinal Microbiota, Facultad de Ciencias de la Vida, Universidad Andrés Bello, Santiago, Chile; ^2^​ Microbiota-Host Interactions & Clostridia Research Group, Facultad de Ciencias de la Vida, Universidad Andrés Bello, Santiago, Chile; ^3^​ Centro de Biotecnología Vegetal, Facultad de Ciencias de la Vida, Universidad Andrés Bello, Santiago, Chile; ^4^​ FONDAP Center for Genome Regulation, Universidad Andrés Bello, Santiago, Chile; ^5^​ Center for Bioinformatics and Integrative Biology, Facultad de Ciencias de la Vida, Universidad Andrés Bello, Santiago, Chile; ^6^​ Department of Clinical Laboratory, Faculty of Medicine, Pontificia Universidad Católica de Chile, Santiago, Chile; ^7^​ Department of Gastroenterology, Faculty of Medicine, Pontificia Universidad Católica de Chile, Santiago, Chile; ^8^​ Facultad de Microbiología & Centro de Investigación en Enfermedades Tropicales (CIET), Universidad de Costa Rica, San José, Costa Rica

**Keywords:** *Clostridium difficile*, B1/NAP1/RT027/ST01 strain, phylogenomics, Latin America, MGEs

## Abstract

*
Clostridium difficile* B1/NAP1/RT027/ST01 has been responsible for outbreaks of antibiotic-associated diarrhoea in clinical settings worldwide and is associated with severe disease presentations and increased mortality rates. Two fluoroquinolone-resistant (FQR) lineages of the epidemic B1/NAP1/RT027/ST01 strain emerged in the USA in the early 1990s and disseminated trans continentally (FQR1 and FQR2). However, it is unclear when and from where they entered Latin America (LA) and whether isolates from LA exhibit unique genomic features when compared to B1/NAP1/RT027/ST01 isolates from other regions of the world. To answer the first issue we compared whole-genome sequences (WGS) of 25 clinical isolates typed as NAP1, RT027 or ST01 in Costa Rica (*n*=16), Chile (*n*=5), Honduras (*n*=3) and Mexico (*n*=1) to WGS of 129 global isolates from the same genotype using Bayesian phylogenomics. The second question was addressed through a detailed analysis of the number and type of mutations of the LA isolates and their mobile resistome. All but two B1/NAP1/RT027/ST01 isolates from LA belong to the FQR2 lineage (*n*=23, 92 %), confirming its widespread distribution. As indicated by analysis of a dataset composed of 154 WGS, the B1/NAP1/RT027/ST01 strain was introduced into the four LA countries analysed between 1998 and 2005 from North America (twice) and Europe (at least four times). These events occurred soon after the emergence of the FQR lineages and more than one decade before the first report of the detection of the B1/NAP1/RT027/ST01 in LA. A total of 552 SNPs were identified across all genomes examined (3.8–4.3 Mb) in pairwise comparisons to the R20291 reference genome. Moreover, pairwise SNP distances were among the smallest distances determined in this species so far (0 to 55). Despite this high level of genomic conservation, 39 unique SNPs (7 %) in genes that play roles in the infection process (i.e. *slpA*) or antibiotic resistance (i.e. *rpoB*, *fusA*) distinguished the LA isolates. In addition, isolates from Chile, Honduras and Mexico had twice as many antibiotic resistance genes (ARGs, *n*=4) than related isolates from other regions. Their unique set of ARGs includes a *cfr*-like gene and *tetM*, which were found as part of putative mobile genetic elements whose sequences resemble undescribed integrative and conjugative elements. These results show multiple, independent introductions of B1/NAP1/RT027/ST01 isolates from the FQR1 and FQR2 lineages from different geographical sources into LA and a rather rapid accumulation of distinct mutations and acquired ARG by the LA isolates.

## Data Summary

This study generated sequencing data for 25 LA isolates and used published data for NAP1/027 isolates from Europe, North America, Australia and Asia [1]. Isolate metadata and accession numbers can be found in Table S1 (available in the online version of this article).

**Impact Statement**

Little is known about the epidemiology of the *C. difficile* B1/NAP1/RT027/ST01 strain outside North America and Europe. Our results expand current knowledge on its dissemination dynamics and microevolution in four countries that span Latin America and justify further surveillance of antibiotic resistance in *C. difficile* from this region.

## Introduction

*Clostridium difficile* is a Gram-positive, strictly anaerobic and spore-forming bacterial pathogen [1]. Up to 50 % of hospitalized patients and about 15 % of healthy adults are normally colonized with *C. difficile* [2], where it can proliferate due to microbiome dysbiosis elicited by antibiotic use, secrete one or more toxins, and thereby trigger diarrhea [3]. *C. difficile* infections (CDI) are nowadays the most common cause of healthcare-associated infections in several countries [4] and have gained notoriety on account of their morbidity, increased mortality [5] and the heavy financial burden that they impose to health systems [6].

A couple of studies published around 2005 reported an unusual increase in the number of CDI cases in North America and Europe [7, 8]. This situation was linked to the spread of a strain classified as B1/NAP1/RT027/ST01 by restriction endonuclease analyses, PFGE, ribotyping and multilocus sequence typing (MLST), respectively [7–9]. Through phylogenomic analyses, it is now known that two lineages of this strain (FQR1 and FQR2) independently acquired resistance to fourth-generation fluoroquinolones in the 1990s [10] by fixation of a Thr82Ile mutation in GyrA [11] and that this mutation facilitated their rapid expansion and transcontinental dissemination from the USA [10]. The so-called FQR1 lineage was transmitted to South Korea and Europe, whereas the FQR2 lineage reached the UK, Europe and Australia [10].

As indicated by reports from Costa Rica [12], Panama [13], Chile [14], Mexico [15], Colombia [16] and Honduras [17], the B1/NAP1/RT027/ST01 strain has been present for at least 10 years in Latin America (LA). However, since nearly all in-depth studies on the molecular epidemiology of this epidemic strain have been conducted in Europe and the USA, the origin, date of introduction, diversity and microevolution of the B1/NAP1/RT027/ST01 strain in LA remains obscure. The antibiotic resistance genotypes of this strain in LA are also unknown, yet it is relevant to study them due to the role of this trait in dissemination and therapy and the distinct manner in which antibiotics that predispose to CDI are prescribed in the region [18, 19]. For instance, there are differences in the use of clindamycin in North America (1.7 % of prescribed antibiotics) and LA (2.7 % of prescribed antibiotics) [18]; a lincosamide antibiotic that has been associated with outbreaks of CDI [20].

To answer these open questions, we generated draft genome sequences (WGS) for 25 B1/NAP1/RT027/ST01 isolates recovered between 2009 to 2016 in Costa Rica, Chile, Honduras and Mexico and analysed this dataset in the context of a global collection of WGS from B1/NAP1/RT027/ST01 isolates using SNP-based analyses, Bayesian phylogenetics, a phylogeographic approach and pangenomics. This study provides insight into the establishment and evolution of the B1/NAP1/RT027/ST01 epidemic strain in LA and assists in the epidemiologic surveillance of *C. difficile *.

## Methods

### Isolates

LA isolates (*n*=25) were recovered by cultivation of loose stool samples pre-treated with 96 % ethanol [21] on Cycloserine-Cefoxitin Fructose Agar (CCFA) or Taurocholate Cycloserine-Cefoxitin Fructose Agar plates (TCCFA). Whereas the former type of agar plates was applied in Costa Rica, Mexico and Honduras, TCCFA was used in Chile. Only samples with positive results for TcdB by immunochromatographic methods (Costa Rica and Honduras), toxigenic culture (Mexico), or the GeneXpert *C. difficile* PCR assay (Chile) were considered for cultivation. Once obtained in pure culture, isolates were identified by a combination of phenotypic and genotypic tests, including colony morphology, UVfluorescence, latex agglutination (Oxoid DR1107), detection of GDH and/or TcdB [22] or PCR-based amplification of *tpi* and toxin genes using primers and conditions reported previously [23]. Isolates from Mexico, Honduras and Costa Rica were typed with a PFGE procedure that was derived from published protocols [24]. Isolates from Chile and Honduras were ribotyped [25] and the resulting band patterns were compared to that obtained with the same procedure for the reference strain R20291 (B1/NAP1/RT027/ST01). Only isolates classified as NAP1 or RT027 were included in the study. With a single exception (a 9-year-old patient from Mexico), all isolates were derived from hospitalized adult patients with diarrhea. Isolate metadata is presented in Table S1.

### Sequence filtering, assembly and annotation

WGS for the 25 LA isolates were obtained through sequencing by synthesis on HiSeq or MiSeq platforms (Illumina). Whereas 16 isolates from Costa Rica were sequenced using paired reads of 100 bp length (5700, 5703, 5705, 5708, 5709, 5710, 5713, 5714, 5718, 5720, 5749, 5758, 5759, 5764, 5765 and 5768), paired reads of ca. 200 bp were generated for one isolate from Mexico (DF11), three isolates from Honduras (HON06, HON10 and HON11) and five isolates from Chile (PUC47, PUC51, PUC99, PUC347 and PUC577). All reads were trimmed at their 3′ end with TRIMMOMATIC v0.36 [26] using a minimum threshold of average quality=Q20 in a window of seven bases. The resulting fastq files were checked visually using FastQC v0.11.7 [27]. For assembly we used Unicycler v0.3.0b using default settings and SPAdes v3.10.1 [28]. Only contigs >200 bp were retained for downstream analyses. For annotation we used Prokka 1.13 and the databases UniProtKB, High-quality Automated and Manual Annotation of Proteins (HAMAP), and Pfam-A [29]. For comparative purposes we downloaded reads for the 151 isolates analysed by He *et al.* in a pioneering study on the global population structure of the NAP1 strain [10] and assembled/annotated them as indicated above. This data was quality-checked using Kraken v2.0.7 [30], SRST2 v0.2.0 [31], bowtie2 v2.3.5 [32] coupled to samtools v1.9 [33], and FastANI [34]. Genomes with contamination, STs different to 1, low coverage (<33X), and/or average nucleotide identity (ANI) values below 99 % were discarded. Assembly statistics for the 25 LA isolates and the 129 WGS from the global population that passed the quality-control checks are provided in Table S1.

### SNP discovery and analyses

SNPs were called with the Bowtie2 mapper in the CFSAN SNP Pipeline v2.1.1 [35], which was run with default options and using a complete genome of *C. difficile* R20291 as a reference (accession number AM180355). Reads with base and map qualities below Q30 and Q13 were excluded, respectively. The minimum variant allele frequency threshold was set to 0.90 and genomic regions with >3 SNPs in a 1000 bp window were filtered out because they might have arisen by recombination [36]. SNPs were annotated and classified based on their potential effects on annotated genes using SnpEff v4.3 [37]. In some cases, the JNet secondary structure prediction tool in the Jpred Server version 4.0.0 [38, 39] was used to visualize and predict whether selected gene variants would change the secondary structure of their translation products.

### Phylogenetic and phylogeographic analyses

beast v1.10.4 was used to obtain estimates of phylogenetic and phylogeographic relationships and node dating using isolate metadata (isolation dates and geographic origin) [10]. To this end, we used the GTR substitution model (chosen with jModelTest v0.1.1 [40]) in combination with an uncorrelated relaxed clock model and the skyline population model, as recommended for global datasets [10]. These tests were carried out with four independent Markov chains, each with a chain length of 100 000 000 states and resampling every 10 000 states. Effective sample size values were >200 for all parameters and convergence and mixing were assessed using Tracer v1.7.1 [41]. Log files were summarized with treeannotator v2.4.8 [42] using 10 % burning, along with maximum clade credibility and node heights at the heights of common ancestors. Trees were plotted with a R script that can be downloaded from the link: https://doi.org/10.6084/m9.figshare.11369871.

### Detection of antibiotic resistance genes (ARGs) and mobile genetic elements (MGEs) with ARGs

ARGs were detected using ariba v2.14.4 [43] and the CARD database v3.0 [44]. Moreover, blastn v2.8.1+ [45] was used to compare contigs with ARGs from the LA WGS collection to contigs from the nearest strains in the Bayesian analysis or from reference strain R20291. This was done to clarify whether the ARGs solely seen in the LA isolates are associated with putative MGEs. Hits were annotated with InterProScan v5.32–71 using default settings [46] and visualized using EasyFig v2.2.2 [47]. Integrative and conjugative elements (ICEs) were identified in the WGS using the blastn function of the ICEberg webserver (db-mml.sjtu.edu.cn/ICEberg/, database version: 2 May 2018) [48].

## Results

### SNP, phylogenomic and phylogeographic analyses

Our mapping approach identified a total of 552 SNPs among the 154 isolates examined. Nearly 86 % of these SNPs are non-synonymous (*n*=476), and 39 from them were solely seen among the LA isolates (Table S2). This subset of isolates was differentiated from those of the global population by 1 to 54 SNPs (Table S3), which is a small distance compared to data from other ribotypes [49]. Isolates PUC347, PUC47 and PUC51 from Chile were distinguished by a mutation in *slpA* that changes the polarity of an amino acid residue (Table S2, Fig. S1). Likewise, seven SNPs in genes for putative metabolic enzymes or a transcriptional anti-terminator differentiated the isolates from Costa Rica (Table S2).

**Fig. 1.**
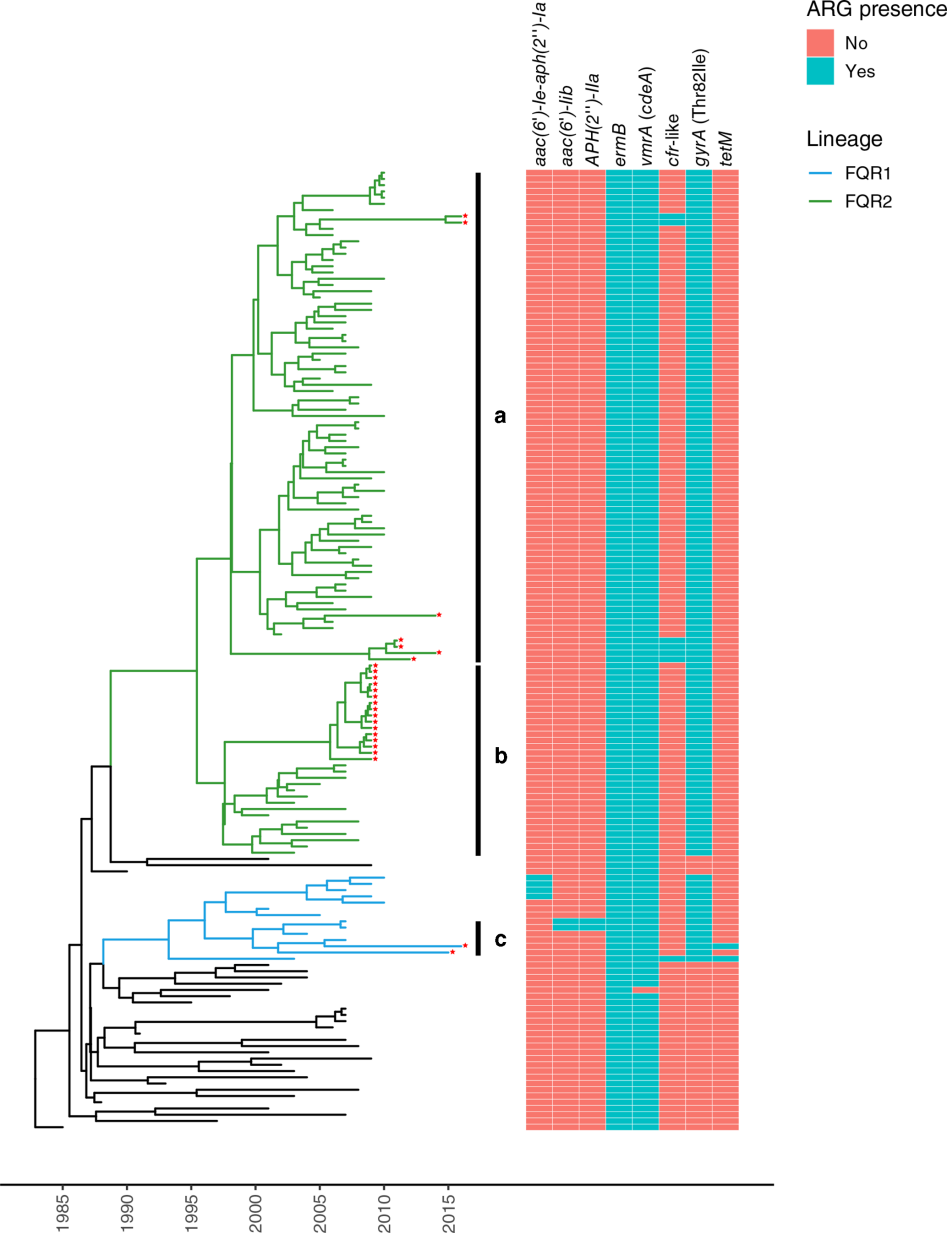
Maximum clade credibility time-scaled tree of 25 *C. difficile* B1/NAP1/RT027/ST01 isolates from Mexico, Honduras, Costa Rica and Chile and 129 isolates of the same genotype from other regions of the world. This tree was derived from a beast analysis of an alignment of 552 SNPs. FQR1 and FQR2 lineages branches are shown in blue or green colour, respectively. A presence (green)/absence (orange) matrix of ARGs and the Thr82Ile mutation in *gyrA* is presented next to the tree. The LA isolates are marked with a red star. Clades a, b and c include LA isolates.

**Fig. 2.**
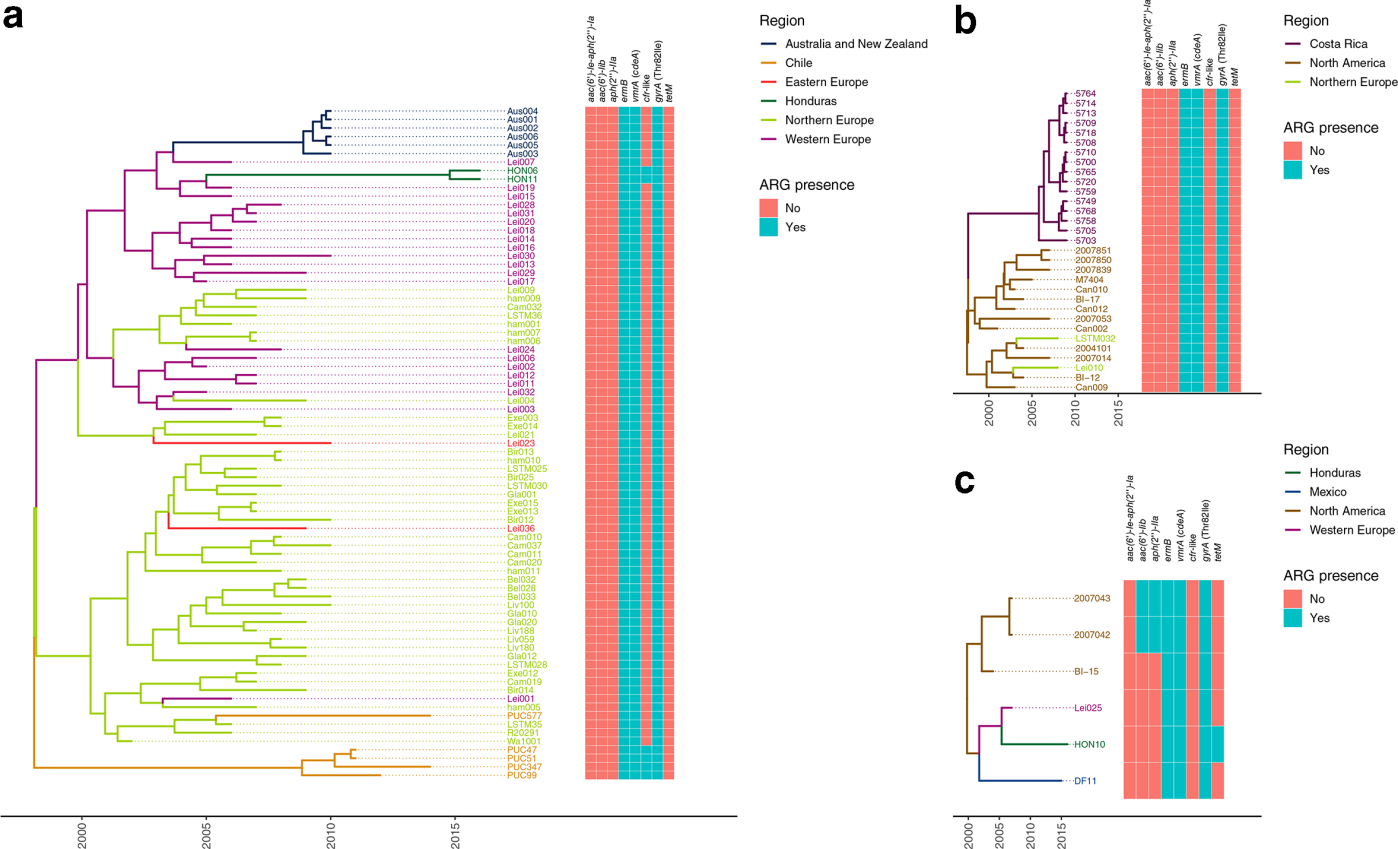
*
C. difficile* B1/NAP1/RT027/ST01 isolates from Mexico, Honduras, Costa Rica and Chile and nearest strains from a global WGS collection. Clades a, b, c from Fig. 1 were enlarged to show LA isolates and their nearest strains in a global collection of B1/NAP1/RT027/ST01 WGS. (a) includes isolates HON06 and HON11 (Honduras) and isolates PUC577, PUC47, PUC51, PUC347 and PUC99 (Chile). (b) includes isolates 5700, 5703, 5705, 5708, 5709, 5710, 5713, 5714, 5718, 5720, 5749, 5758, 5759, 5764, 5765 and 5768 (Costa Rica), (c) includes isolates HON10 (Honduras) and DF11 (Mexico). Branch colours indicate the geographic origin of the isolates. A presence (green)/absence (orange) matrix of ARGs and the Thr82Ile mutation in *gyrA* is presented next to the tree.

Our SNP-based Bayesian (Figs 1 and 2) and maximum-likelihood (ML) (Fig. S2) phylogenetic analyses succeeded in delimitating the expected FQR1 and FQR2 lineages and assigned the same isolates to each group. Isolates from the FQR2 lineage (*n*=23/25), and to a much lesser extent, from the FQR1 lineage (*n*=2/25) were found to circulate in the four LA countries studied (Figs 1 and 2 and S2). All isolates from Costa Rica formed a clade with a posterior probability=1 and a maximum SNP distance of 7 (Fig. 2b, Table S3). Hence, they likely represent a single outbreak clone.

**Fig. 3.**
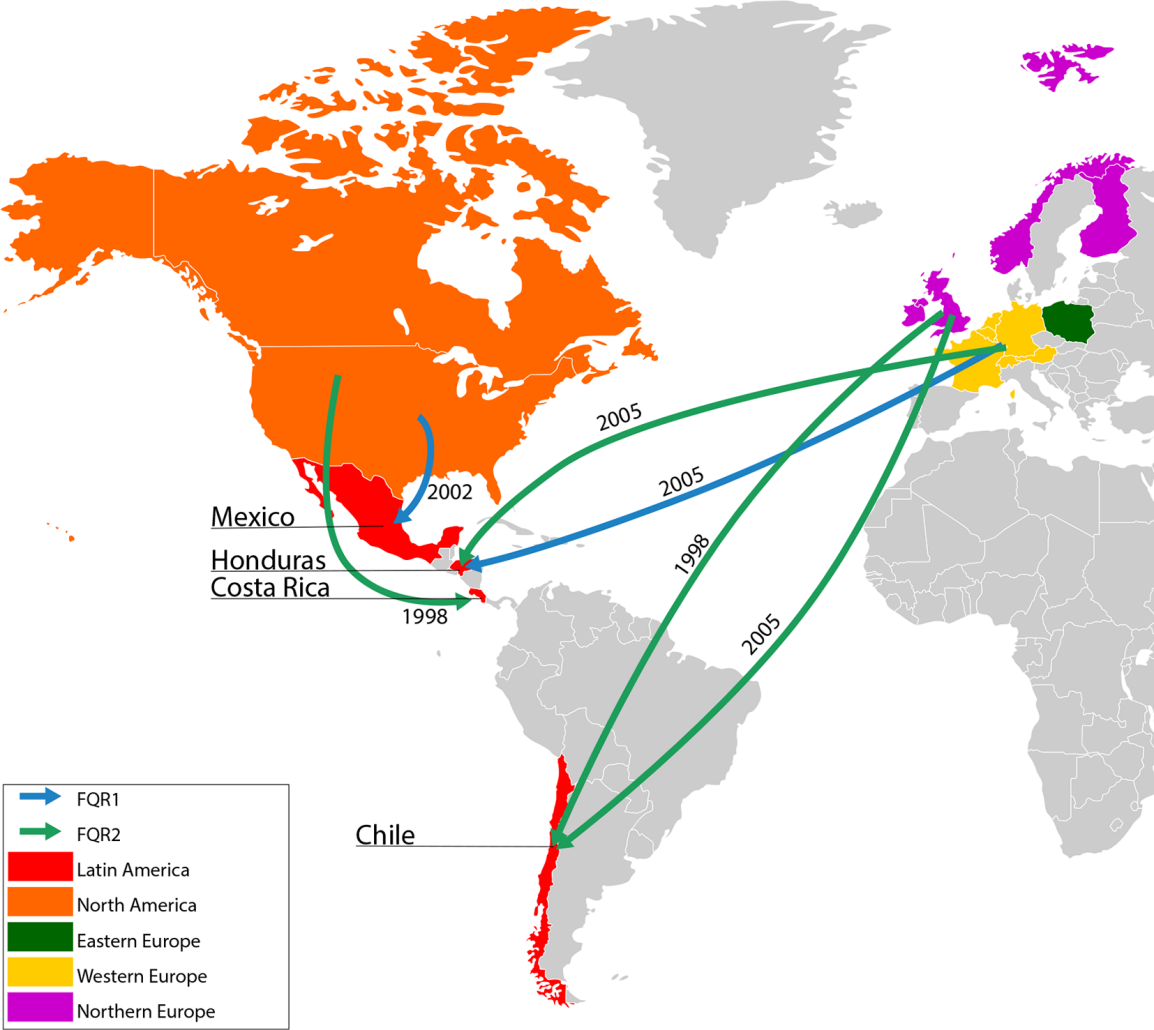
Possible introduction events of *C. difficile* B1/NAP1/RT027/ST01 isolates to Mexico, Honduras, Costa Rica and Chile. Coloured arrows indicate the most probable migration route of FQR1 (blue) or FQR2 (green) isolates to four LA countries along with its year of entrance (median estimates of 95 % highest posterior density intervals). Only countries for which isolates were available appear in colour.

We noted topological differences in the vicinity of the FQR2 LA isolates in the Bayesian- and ML-based trees (Figs 2 and S2). In this regard, we favoured the former method due to low branch support values obtained by ML and because it allows prediction of transmission events. According to our small dataset, FQR2 isolates possibly entered Honduras (once), Costa Rica (once) and Chile (twice) from North America (Costa Rica) or Western and Northern Europe (Honduras and Chile, Fig. 3). This lineage was likely introduced around 1998 [95 % highest posterior density interval (HPD): 1994–2000], when *C. difficile* Can002 (USA, 12 SNPs apart from 5705, 5758 and 5768) or a closely related strain entered Costa Rica [posterior state probability (PSP): 98.5 %]. Thereafter, *C. difficile* Lei019 (Western Europe, 7–8 SNPs apart from HON06 and HON11) or a closely related strain was introduced into Honduras (PSP: 99.1 %) in 2005 (95 % HPD: 2003–2006). Our data also supports two introductions of FQR2 isolates from Northern Europe to Chile around 1998 (95 % HPD: 1994–2001, PSP: 70.0 %) and 2005 (95 % HPD: 2004–2006, PSP: 99.9 %) (Fig. 3). The nearest strains to the PUC strains in our Bayesian phylogeographic analysis are *C. difficile* Wa1001 (apart from PUC99 and from PUC51, PUC47 and PUC347 by 4 and 8 SNPs, respectively) and *C. difficile* LSTM35, which is only two SNPs apart from PUC557).

FQR1 strains were likely imported into LA when strains BI-15 and Lei025, which are 14 and 9 SNPs apart from DF11 and HON10, or closely related strains were transferred to Mexico from North America around 2002 (95 % HPD: 1998–2005, PSP: 58.5 %) or to Honduras from Western Europe around 2005 (95 % HPD: 2003–2007, PSP: 87.4 %) (Fig. 3).

### Mutations and laterally acquired genes implicated in antibiotic resistance

All LA isolates possessed the Thr82Ile mutation in the *gyrA* gene that established the FQR lineages and explains their high-level fluoroquinolone resistance (Fig. 2). In addition, the FQR1 isolates DF11 and HON10 also showed two mutations in *rpoB* (Table S2) that have been implicated in rifampicin resistance (Arg505Lys and Ile548Met) [50] and DF11 had a mutation in *fusA* (Table S2) that has been linked to resistance to fusidic acid (Glu117Lys) [10, 51]. We did not confirm the role of these mutations in resistance experimentally.

As to acquired ARGs, all but one of the 154 isolates studied (Can001) carry the multidrug and toxic compound extrusion (MATE) multidrug efflux transporter *vmrA*, also known as *cdeA,* and *ermB* (Figs 1 and 2). Unlike isolates from the global population, LA isolates HON06, HON11, PUC47, PUC51, PUC99 and PUC347 have a *cfr*-like gene and HON10 also carries *tetM* (Fig. 2). These genes are expected to confer them with resistance to phenicols, lincosamides, pleuromutilins, and streptogramin A [52], or tetracycline [53], respectively.

The genomic localization of the *cfr*-like and *ermB* genes detected in the HON and PUC isolates differed. HON isolates have them separately located in two putative MGEs, whereas the PUC isolates carry them in a single genetic structure of ca. 45 kb (Figs S3–S5).

The putative MGE of the HON isolates with a *cfr*-like gene shows >98 % of sequence identity to Tn*6218* [54] (Fig. S6). On the other hand, their putative MGE with *ermB* is likely an ICE with genes for an integrase/recombinase (IPR010998), an excisionase (IPR015122), the conjugative transposon protein TcpC (IPR024735), and a protein from the TcpE family (IPR025608) (Fig. S4). These two putative MGEs are absent in strain Lei019, which is the closest relative to the HON isolates in our pairwise SNP distance matrix (seven–eight SNPs apart, Table S3, Figs S3–S4).

The putative MGE of the PUC isolates with the *cfr*-like gene and *ermB* is not present in strains R20291 and Wa1001 (Fig. S5), which were identified as their nearest strains in our SNP analyses (four–eight SNPs apart; Table S3). This putative MGE is likely an ICE, for it has genes for an integrase/recombinase (IPR010998), a relaxase of the MobA/VirD2 type (IPR005094), a transposon-transfer assisting protein (IPR025468), a type IA DNA topoisomerase, (IPR000380), and the type-IV secretion system protein TraG/VirD4 (IPR003688). Only 1135 bp of this putative MGE gave hits with elements deposited in the ICEberg database, yet a similar search in the NCBI non-redundant database matched a 30 kb genomic fragment of *C. difficile* CD161; a strain that lacks *cfr*-like genes (Fig. S7).

The FQR1 isolates from LA also carry putative ICEs with ARG. For instance, the *ermB* gene of HON10 was found in a MGE with genes for an integrase/recombinase (IPR010998), the conjugative transposon protein TcpC (IPR024735), and a protein from the TcpE family (IPR025608) (Fig. S8). This isolate also has *tetM* (Fig. 2c), but in a second putative ICE with genes for an integrase/recombinase (IPR010998), an excisionase (IPR015122), and the conjugative transposon protein TcpC (IPR024735) (Fig. S9). The location of these two putative MGEs in the nearest strain from the phylogenetic reconstruction could not be determined due to the lack of flanking genes in the contigs.

In DF11, *ermB* was also detected in a putative ICE (Fig. S10). This element contains genes for an integrase/recombinase (IPR010998), an excisionase (IPR015122), the conjugative transposon protein TcpC (IPR024735), and a protein from the TcpE family (IPR025608). 2007042, the closest strain to DF11 in our SNP analyses (12 SNPs apart; Table S3) lacks this potential ICE (Fig. S10).

Finally, the *ermB* ^+^ MGEs of HON06, HON10, HON11 partially resemble CTn*1* and CTn*7* of CD630 (Fig. S11). In DF11 the *ermB* ^+^ MGE shows an insertion of a group-II intron and in HON10 *ermB* is inverted (Fig. S12). Though these putative MGEs have nearly identical sequences, they are flanked by different adjacent genes (Fig. S3–12). Detailed annotations for all putative MGEs described in this section are available at https://doi.org/10.6084/m9.figshare.11369658.

## Discussion

This work expands current knowledge on the genomic variation and microevolution of the *C. difficile* B1/NAP1/RT027/ST01 strain by providing new insights into its spread to four LA countries and how it has evolved through the incorporation of exclusive SNPs and ARG genes into novel putative MGEs.

We report circulation of *C. difficile* isolates from both FQR1 and FQR2 lineages in LA and determine that the first transmission event occurred 13 years before isolation of the B1/NAP1/RT027/ST01 strain in Latin America was reported for the first time. This time lapse likely explains why all 25 LA isolates exhibited unique SNPs and acquired putative MGEs not present in the nearest strains from the global collection analysed.

Although the number of isolates investigated is far from being representative of all CDI cases in LA (*n*=25), our set of isolates is the most comprehensive collection of B1/NAP1/RT027/ST01 isolates sequenced to date from this region of the world. This small sample size could explain why we did not register transfer events between LA countries, as opposed to similar studies that considered two to four times more genomes [10, 55, 56].

Most introductions corresponded to isolates from the FQR2 lineage, which has been recognized as the most widely distributed in the world [10]. However, newer studies report that the prevalence of the FQR1 and FQR2 lineages is similar in North America [56]. The most recent common ancestor analysis performed showed that the B1/NAP1/RT027/ST01 strain arrived in LA between 1998 and 2005 from North America (FQR1 and FQR2), Western Europe (FQR1 and FQR2) and Northern Europe (FQR2), confirming the rapid global dissemination of the FQR lineages after their emergence [10].

Previous studies have identified ca. 500 SNPs among collections of 151 and 57 *C. difficile* B1/NAP1/RT027/ST01 isolates [10, 55]. Despite having used different software pipelines, the total number of SNPs called (*n*=552) is comparable to the figure reported by He *et al.* (*n*=536). This result, together with the rather small pairwise SNP distances calculated, confirms that the core genome of the B1/NAP1/RT027/ST01 strain is highly conserved [57].

Our Bayesian and ML analyses classified the He *et al.* isolates as expected and with identical results, adding confidence to our estimates [10]. We are aware of inconsistencies in the distribution of the FQR2 isolates in both types of trees. This situation may arise when the SNP matrix used for computation does not contain strong phylogenetic signals [58]. Irrespective of this caveat, nearly all our inferences of transmission events were linked to posterior probabilities ≥70 %, which are usual value in studies of other human pathogens [59].

The LA isolates showed SNPs and acquired ARGs not seen in isolates from North America, Europe, Australia or Asia. As to the SNPs called, we noted a variation in *slpA*, which encodes the major S-layer precursor protein [60], and whose product participates in adherence [61] and activates the innate and adaptive immune system from the host [62].

All LA strains carry the Thr82Ile mutation in the *gyrA* gene that confers high-level resistance against fluoroquinolones, consistent with this mutation being a key element in the B1/NAP1/RT027/ST01 epidemic lineage [10, 63]. Besides this SNP related to antibiotic resistance, a double substitution was found in the gene for RpoB (Arg505Lys and Ile548Met). These rifampicin-resistance markers [50] were seen in all LA isolates from the FQR1 lineage and in isolates 2007042, 2007043, Lei025, BI-15, BI-13, 2006439 and 2004102 from the global population. The presence of this double substitution in the FQR1 lineage has not been previously reported. Similarly, this study is the first to report a Glu117Lys substitution in *fusA* in the B1/NAP1/RT027/ST01 strain. These findings expand the repertoire of SNPs associated with antibiotic resistance found in this epidemic strain.

We also observed the presence of putative ICEs carrying ARGs in eight isolates, namely HON06, HON10, HON11, PUC47, PUC51, PUC99, PUC347 and DF11. All but one of these elements show over 90 % sequence identity to ICEs included in the ICEberg database and some of them were found in different genomic coordinates. Therefore, we anticipate that they are functional and hypothesize that they were acquired through transposition or conjugation events once the isolates entered LA. Although *cfr*-like genes, *ermB* and *tetM* have already been found in *C. difficile* from other countries [17, 64, 65] we describe here for the first time the presence of a *cfr*-like gene linked to *ermB* in a putative ICE of ca. 46 kb (Fig. S5). This *cfr*-like gene has been recently identified as *cfr*(B) [66]. Additionally, we report new combinations of ARGs in a single B1/NAP1/RT027/ST01 isolate. These results were obtained despite the limited number of isolates analysed. Thus, expanding the repertory of isolates could provide a more detailed insight into the genetic organization and diversity of ARGs within MGEs and their insertion sites in the *C. difficile* genome. Likewise, further work with a larger sample size will provide more insight into transmission of the *C. difficile* B1/NAP1/RT027/ST01 strain among and within LA countries.

Phenotypic assays are required to validate the role of the SNPs and acquired genes in antibiotic resistance. In this context, given that *C. difﬁcile* exchanges DNA with intestinal Firmicutes [54], and possibly with many other members of the complex metagenome with which it coexists [15, 17, 56, 60, 67, 68], our pangenomic analyses justify further surveillance of antimicrobial resistance in this pathogen in LA and the world.

In conclusion, we found that both FQR1 and FQR2 *C. difficile* B1/NAP1/RT027/ST01 lineages were present in our sample cohort and that they emerged in the LA countries sampled earlier than previously thought. We also detected that these lineages entered Costa Rica, Chile, Honduras and Mexico from different geographical locations and demonstrate that B1/NAP1/RT027/ST01 isolates from these countries are prone to acquire distinct SNPs and genes implicated in antibiotic resistance.

## Data Bibliography

1. He M., Miyajima F., Roberts P., Ellison L., Pickard D.J. *et al*. Emergence and global spread of epidemic healthcare-associated *Clostridium difficile*. *Nat. Genet.* 2013;45(1):109-113.

## Supplementary Data

Supplementary material 1

Supplementary material 2
