# Supplementary material for: Origin, genomic diversity and microevolution of the Clostridium difficile B1/NAP1/RT027/ST01 strain in Costa Rica, Chile, Honduras and Mexico
Source: Microb Genom. 2020 Mar 16;6(5):e000355. doi: 10.1099/mgen.0.000355 (PMC7371124; doi:10.1099/mgen.0.000355)
Supplement: Supplementary material 1 [file mgen-6-355-s001.pdf]

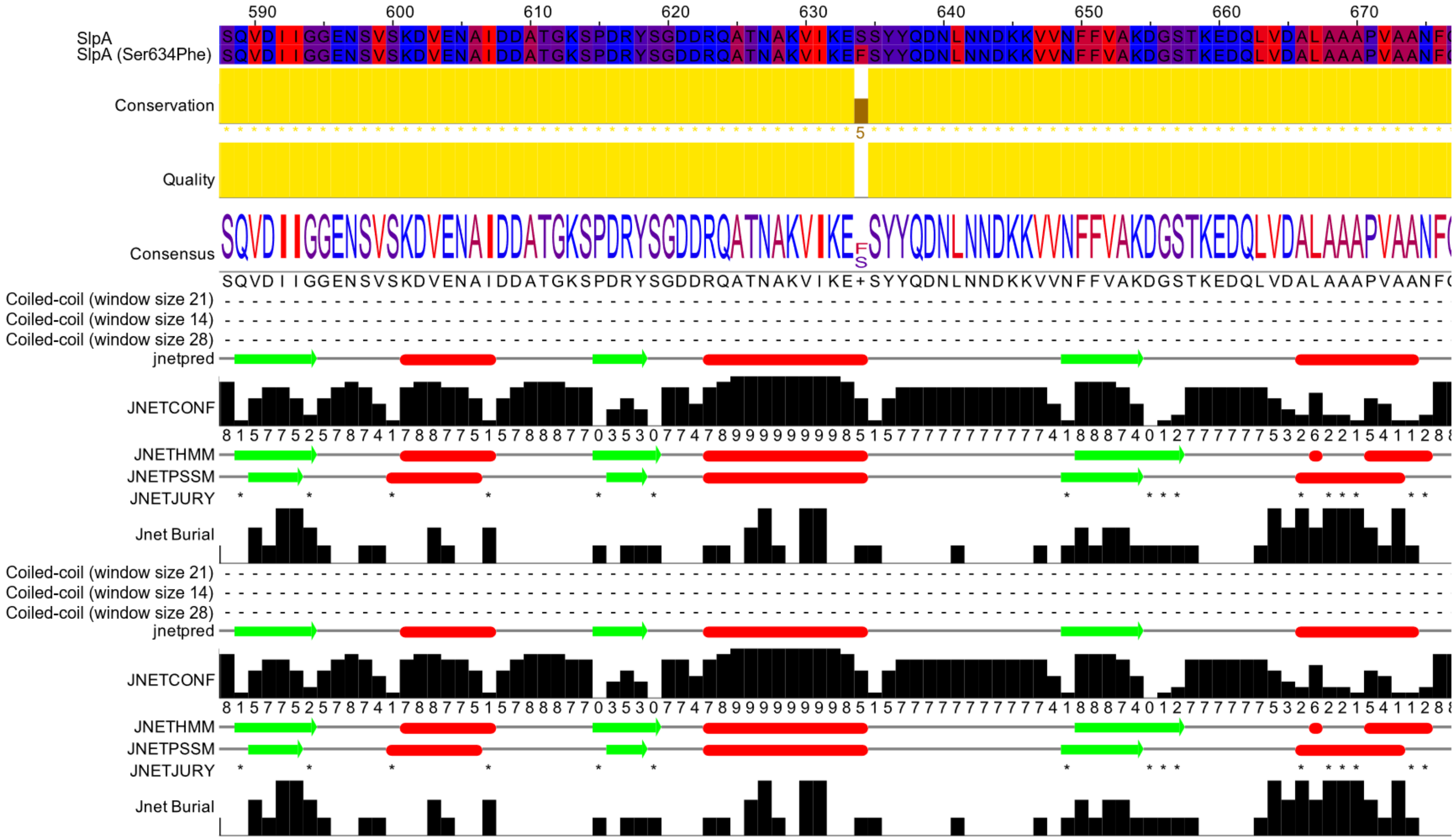

Supplementary Figure 1. Comparison of the amino acidic sequence and the secondary structure predicted by Jpred for a reference SlpA sequence and the SlpA sequence with the Ser634Phe mutation. The annotation includes coiled-coil predictions for three windows sizes; Red and blue tubes indicate α-helices and β-sheets, respectively. JNetCONF is the confidence estimate for the prediction. JNetHMM and JNETPSSM base their predictions on HMM- or PSSM-profiles, respectively. JNETJURY indicates with an '*' positions at which all prediction outputs disagree. JNet burial predict solvent accessibility levels (the higher the bar the lesser level of accessibility).

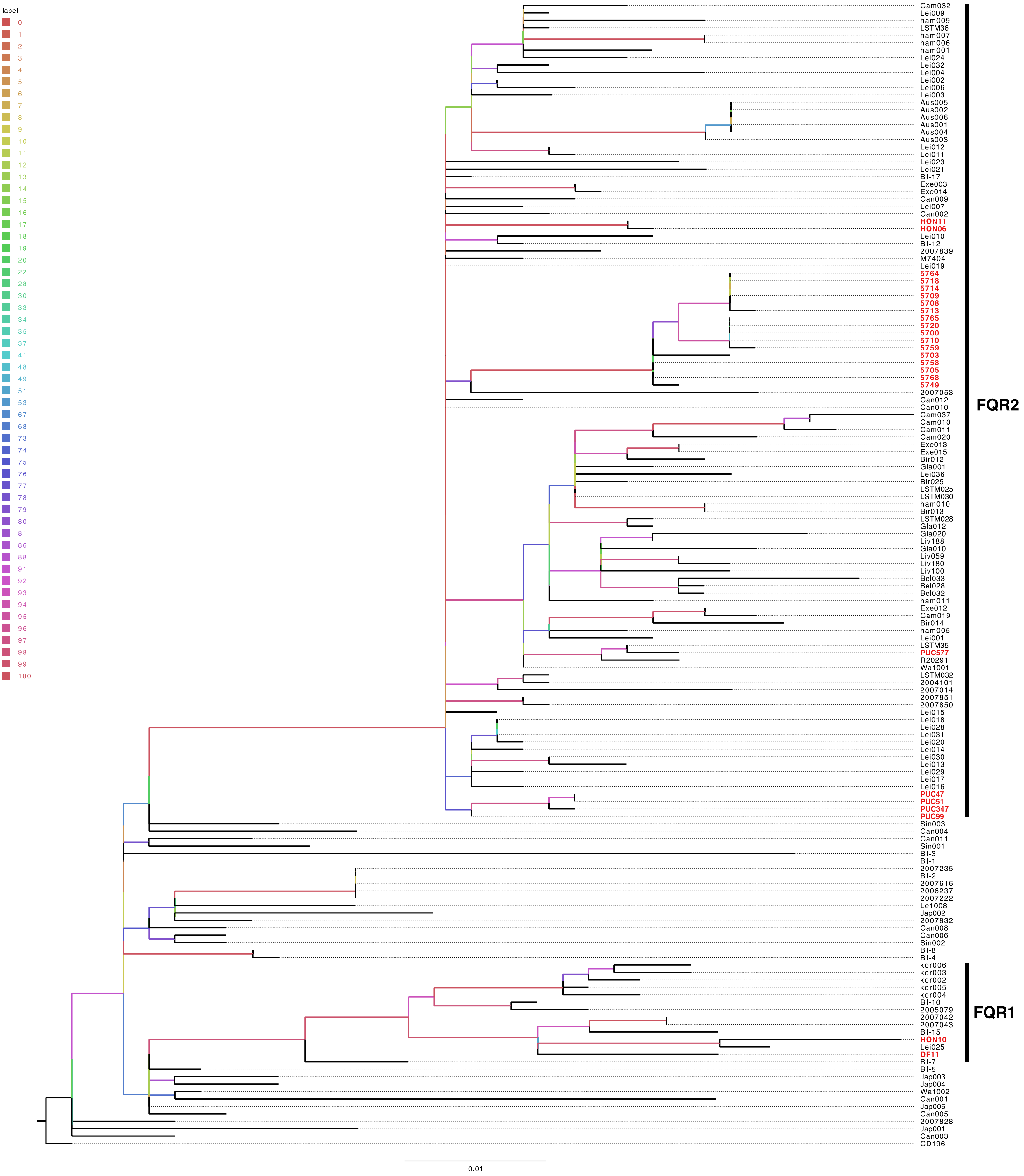
label
0
1
2
3
4
5
6
7
8
9
10
11
12
13
14
15
16
17
18
19
20
22
28
30
33
34
35
37
41
48
49
51
53
67
68
73
74
75
76
77
78
79
80
81
86
88
91
92
93
94
95
96
97
98
99
100
Cam032
Lei009
ham009
LSTM36
ham007
ham006
ham001
Lei024
Lei032
Lei004
Lei002
Lei006
Lei003
Aus005
Aus002
Aus006
Aus001
Aus004
Aus003
Lei012
Lei011
Lei023
Lei021
BI-17
Exe003
Exe014
Can009
Lei007
Can002
HON11
HON06
Lei010
BI-12
2007839
M7404
Lei019
5764
5718
5714
5709
5708
5713
5765
5720
5700
5710
5759
5703
5758
5705
5768
5749
2007053
Can012
Can010
Cam037
Cam010
Cam011
Cam020
Exe013
Exe015
Bir012
Gla001
Lei036
Bir025
LSTM025
LSTM030
ham010
Bir013
LSTM028
Gla012
Gla020
Liv188
Gla010
Liv059
Liv180
Liv100
Bel033
Bel028
Bel032
ham011
Exe012
Cam019
Bir014
ham005
Lei001
LSTM35
PUC577
R20291
Wa1001
LSTM032
2004101
2007014
2007851
2007850
Lei015
Lei018
Lei028
Lei031
Lei020
Lei014
Lei030
Lei013
Lei029
Lei017
Lei016
PUC47
PUC51
PUC347
PUC99
Sin003
Can004
Can011
Sin001
BI-3
BI-1
2007235
BI-2
2007616
2006237
2007222
Le1008
Jap002
2007832
Can008
Can006
Sin002
BI-8
BI-4
kor006
kor003
kor002
kor005
kor004
BI-10
2005079
2007042
2007043
BI-15
HON10
Lei025
DF11
BI-7
BI-5
Jap003
Jap004
Wa1002
Can001
Jap005
Can005
2007828
Jap001
Can003
CD196
FQR2
FQR1
0.01

Supplementary Figure 2. Maximum-likelihood phylogenetic tree for 154 *C. difficile* B1/NAP1/RT027/ST01 isolates. Based on an alignment of 552 SNPs. Branch colors indicate the boostrap value of each node. LA isolates are highlighted in red. FQR lineages are marked with a vertical black line.

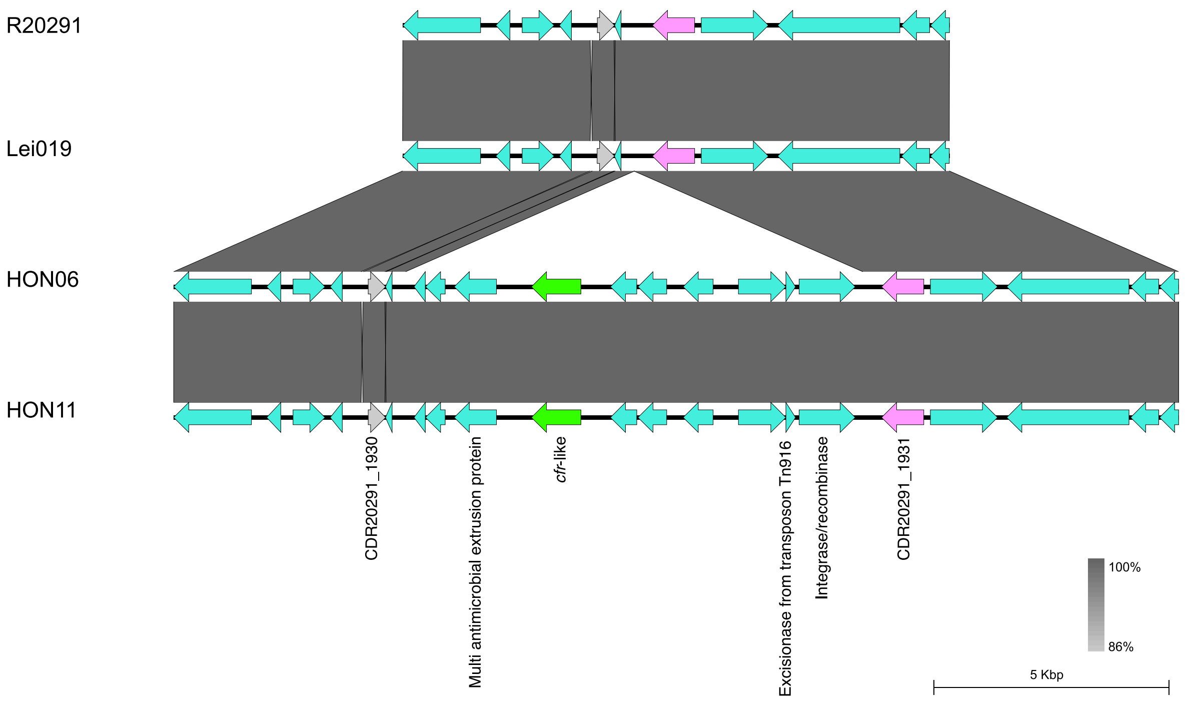

Supplementary Figure 3. Insertion site of a putative MGE with a *cfr*-like gene acquired by isolates HON06 and HON11.

This element is absent in the closest strain from the SNP analyses (Lei019; 8 and 7 SNPs apart from HON06 and HON11, respectively) and the reference strain R20291. It appears inserted between CDS for a putative phage regulatory protein (CDR20291_1930, grey arrow) and a transcriptional regulator from the AraC-family (CDR20291_1931, pink arrow). The *cfr*-like gene is shown as a green arrow. A grayscale indicates the level of nucleotide sequence identity. InterProScan annotations related to MGEs are shown at the bottom of the annotated HON11 sequence.

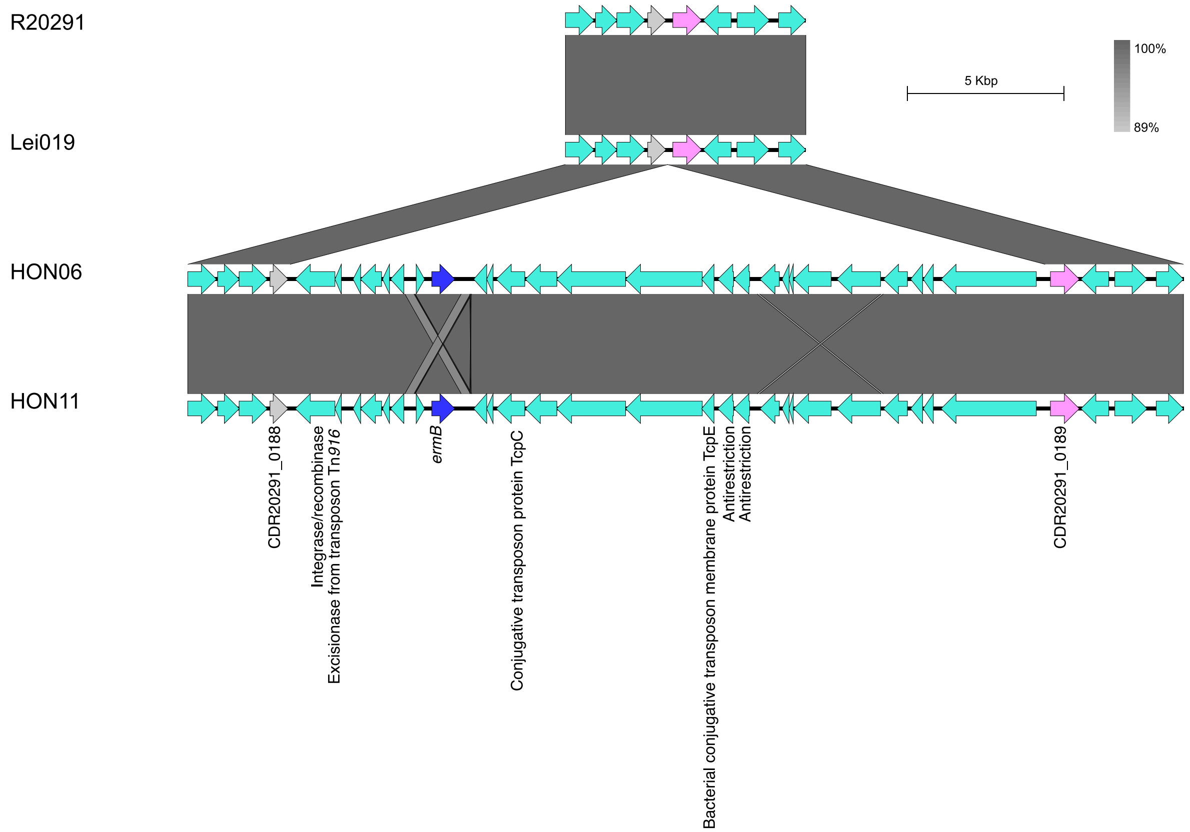

Supplementary Figure 4. Insertion site of a putative *ermB*$^+$ MGE acquired by isolates HON06 and HON11.

This element is absent in the closest strain from the SNP analyses (Lei019; 8 and 7 SNPs apart from HON06 and HON11, respectively) and the reference strain R20291. It appears inserted between CDS for an orotate phosphoribosyltransferase (CDR20291_0188, grey arrow) and a 2-dehydropantoate 2-reductase (CDR20291_0189, pink arrow). The *ermB* gene is shown as a blue arrow. A grayscale indicates the level of nucleotide sequence identity. InterProScan annotations related to MGEs are shown at the bottom of the annotated HON11 sequence.

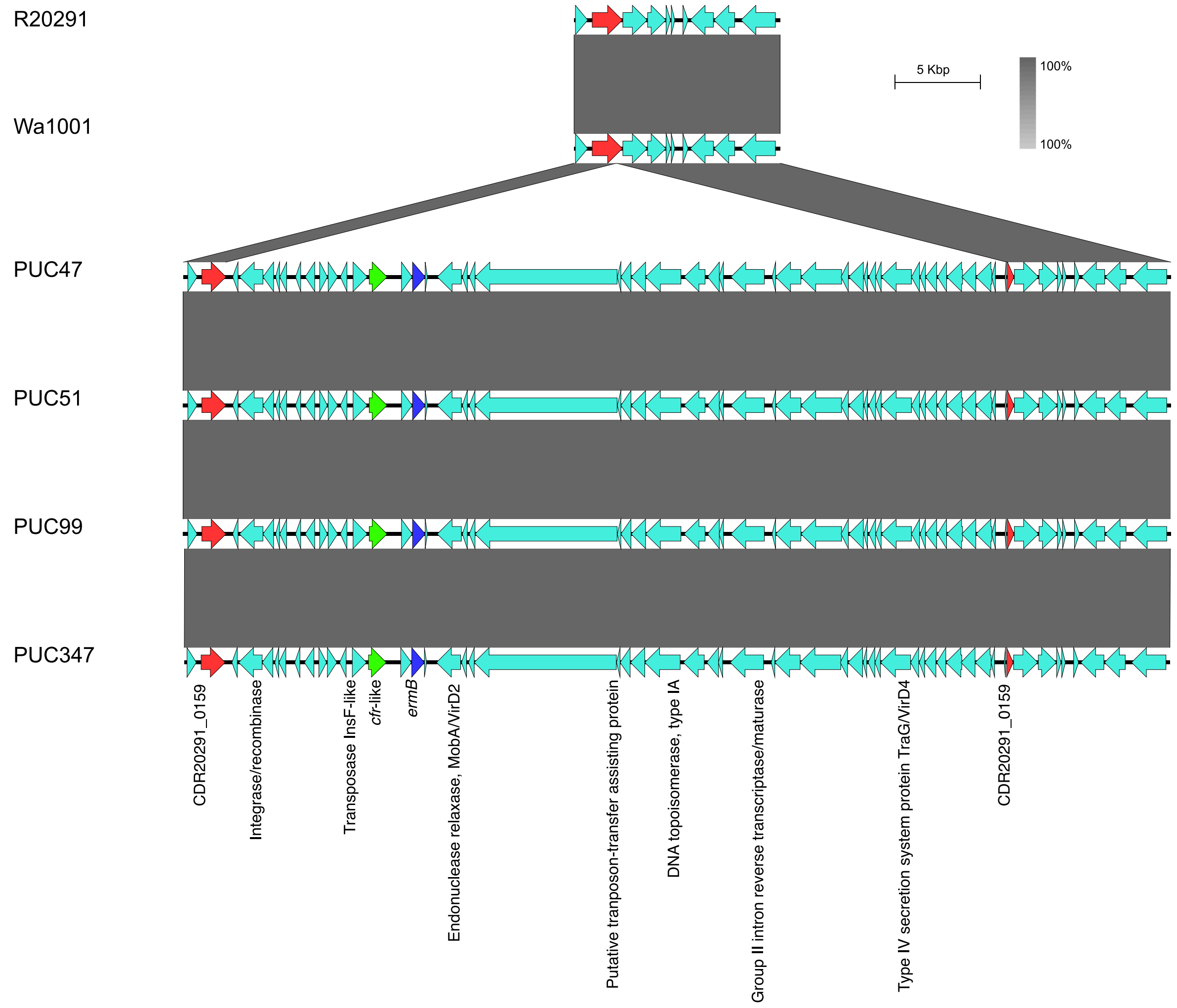

Supplementary Figure 5. Insertion site of a putative MGE positive for *ermB* and a *cfr*-like gene acquired by isolates PUC47, PUC51, PUC99 and PUC347.
This element is absent in the closest strain from the SNP analyses (Wa1001; 4-8 SNPs apart from PUC47, PUC51, PUC99 and PUC347) and the reference strain R20291. It appears inserted into the CDS for a putative lantibiotic ABC transporter (CDR20291_0159, red arrow). The *cfr*-like gene and *ermB* are shown as green and blue arrows, respectively. A grayscale indicates the level of nucleotide sequence identity. InterProScan annotations related to MGEs are shown at the bottom of the annotated PUC347 sequence.

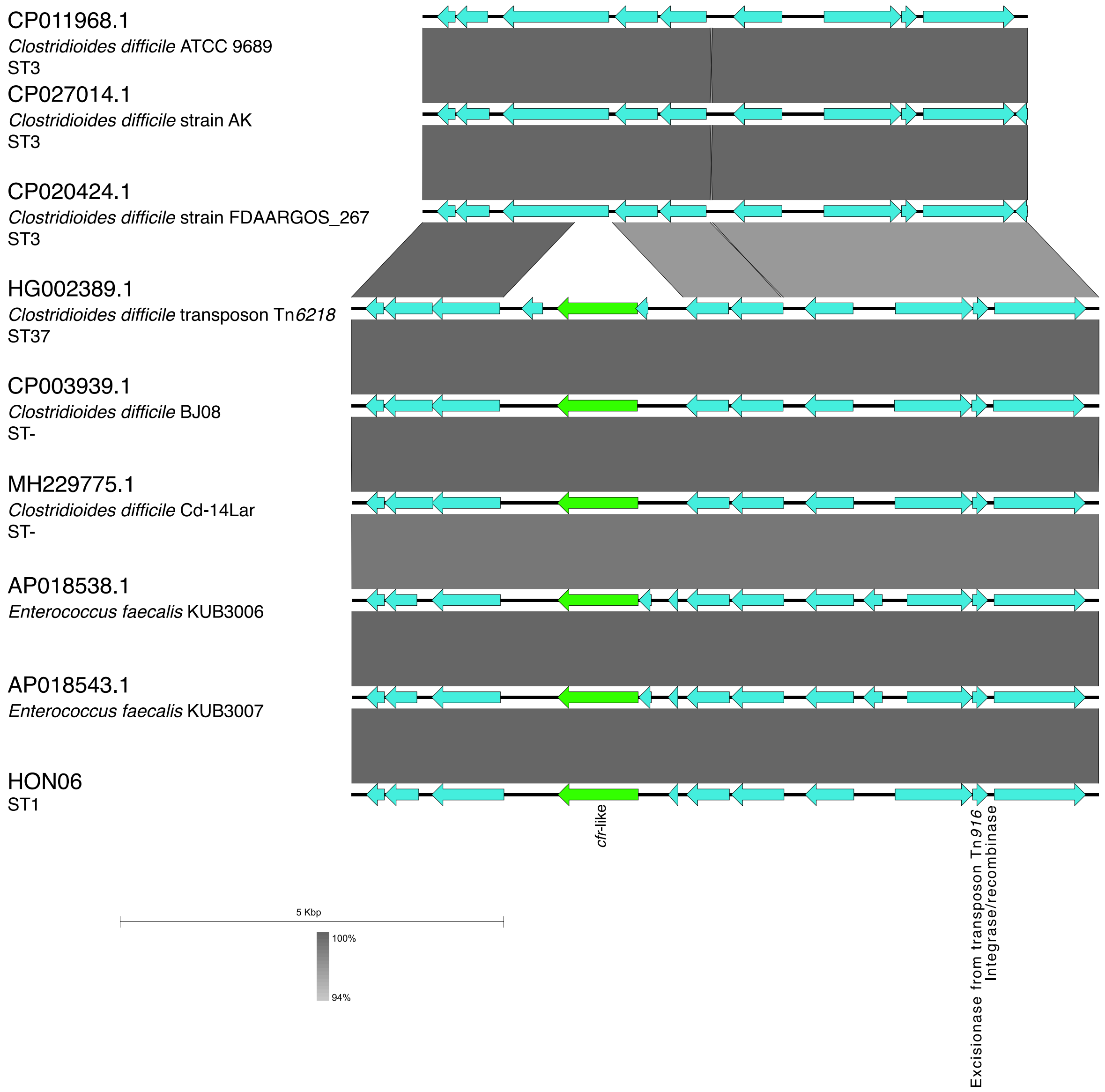

Supplementary Figure 6. Comparison of genomic sequences from various Firmicutes and the putative MGE with a *cfr*-like gene detected in isolates HON06 and HON11.
The *cfr*-like gene detected in isolates HON06 and HON11 (green arrow) is included in identical genomic fragments that resemble ICEs deposited in the ICEberg database. Only the sequence of HON06 is shown in the figure. Accession numbers and strain names are shown at the left. STs are shown for all *C. difficile* strains. InterProScan annotations related to MGEs are shown at the bottom of the annotated HON06 sequence.

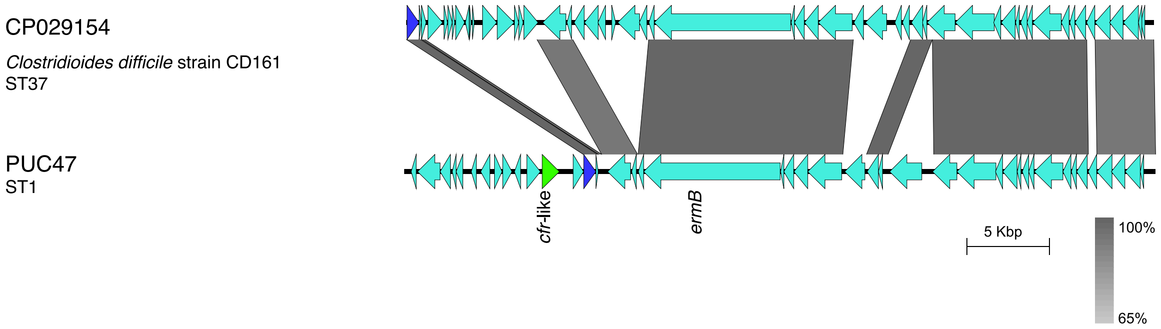

Supplementary Figure 7. Comparison of the putative MGE with a *cfr*-like gene and *ermB* identified in the PUC isolates to a genomic fragment from *C. difficile* CD161

The *cfr*-like gene and *ermB* are shown as green and blue arrows, respectively. Only the sequence of PUC47 is shown. The genomic fragment of CD161 (ST37) was the best hit found by BLASTn in the non-redundant NCBI nucleotide database. InterProScan annotations related to MGEs are shown at the bottom of the annotated PUC47 sequence.

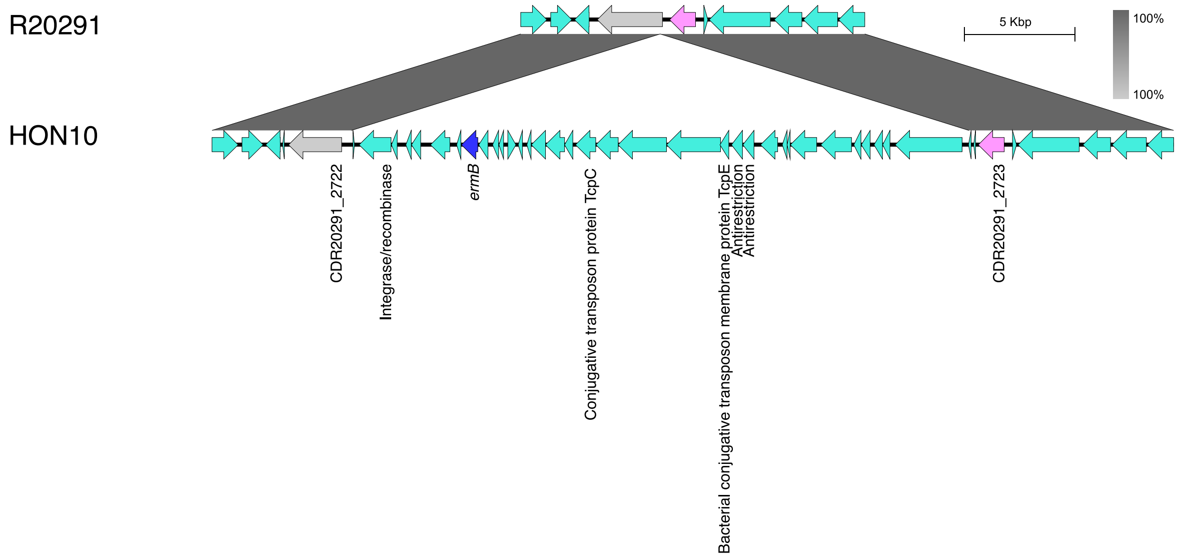

Supplementary figure 8. Insertion site of the putative *ermB*⁺ MGE acquired by isolate HON10.

This element is absent in the reference strain R20291. It appears inserted into the CDS for a putative collagen-binding protein (CDR20291_2722, grey arrow). The *ermB* gene is shown as a blue arrow. A grayscale indicates the level of nucleotide sequence identity. InterProScan annotations related to MGEs are shown at the bottom of the annotated HON10 sequence.

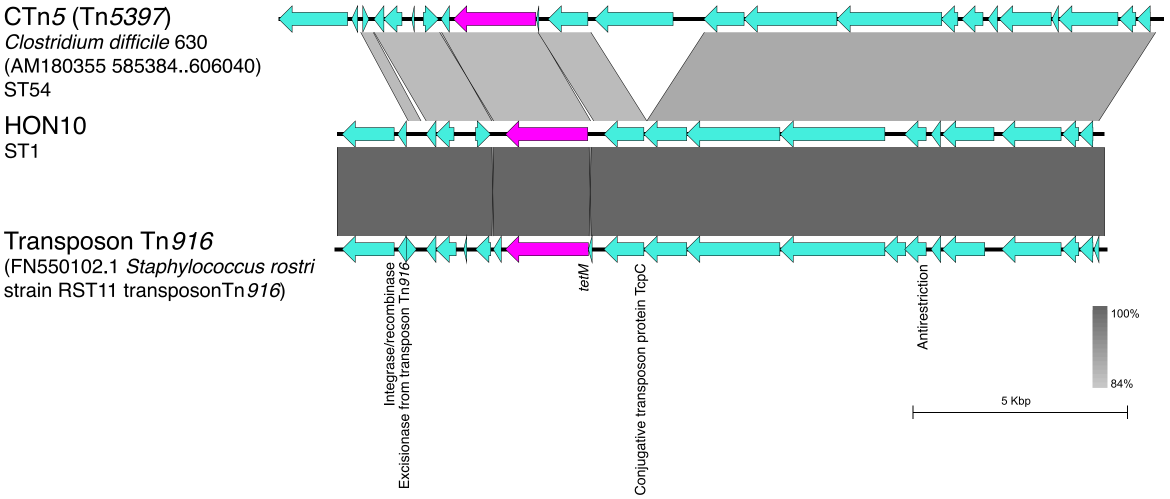

Supplementary Figure 9. Relatedness of the putative *tetM*+ MGE identified in isolate HON10 to Tn*5397* and Tn*916*.

A BLASTn search in the ICEBerg database revealed that the putative MGE of isolate HON10 with *tetM* (pink arrow) is related to well-known tetracycline resistance transposons. A grayscale indicates the level of nucleotide sequence identity. InterProScan annotations related to MGEs are shown at the bottom of the annotated Tn*916* sequence.

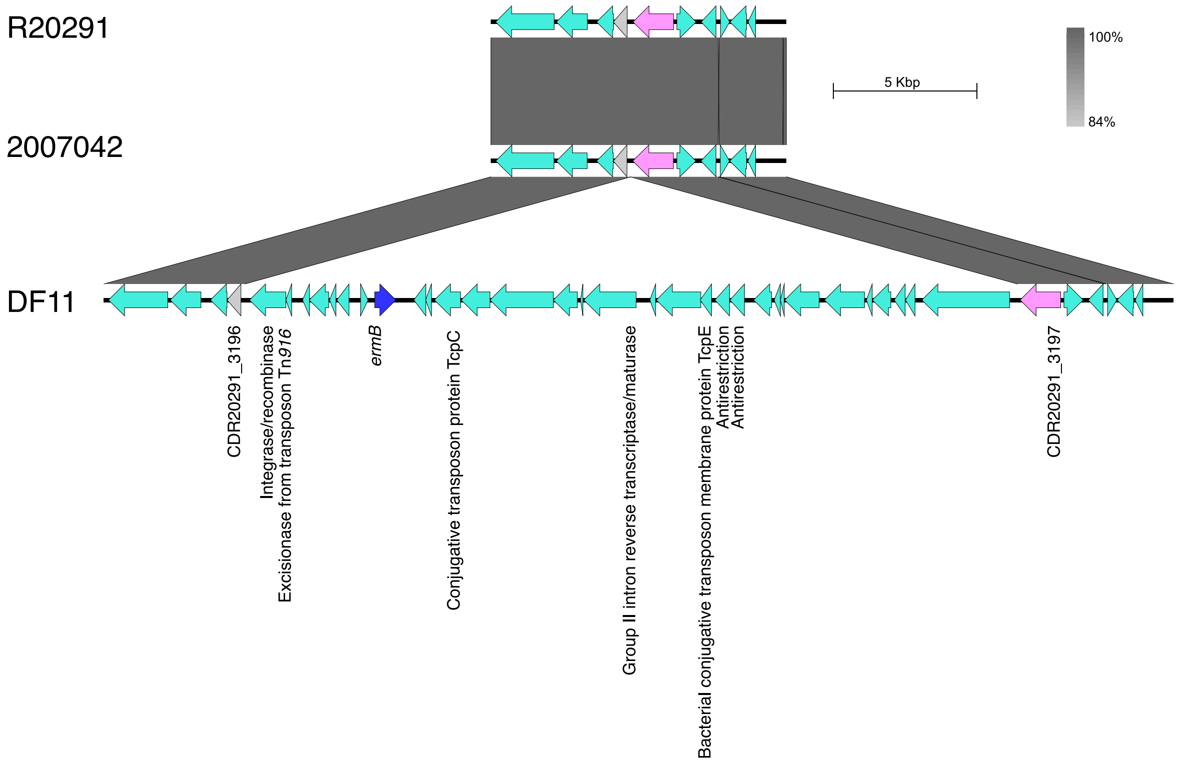

Supplementary Figure 10. Insertion site of a putative *ermB*+ MGE acquired by isolate DF11.

This element is absent in the closest strain from the SNP analyses (2007042; 12 SNPs apart from DF11) and the reference strain R20291. It appears inserted between the CDS for an AraC-family transcriptional regulator (CDR20291_3196, grey arrow) and a GntR-family transcriptional regulator (CDR20291_3197, pink arrow). The *ermB* gene is shown as a blue arrow. A grayscale indicates the level of nucleotide sequence identity. InterProScan annotations related to MGEs are shown at the bottom of the annotated DF11 sequence.

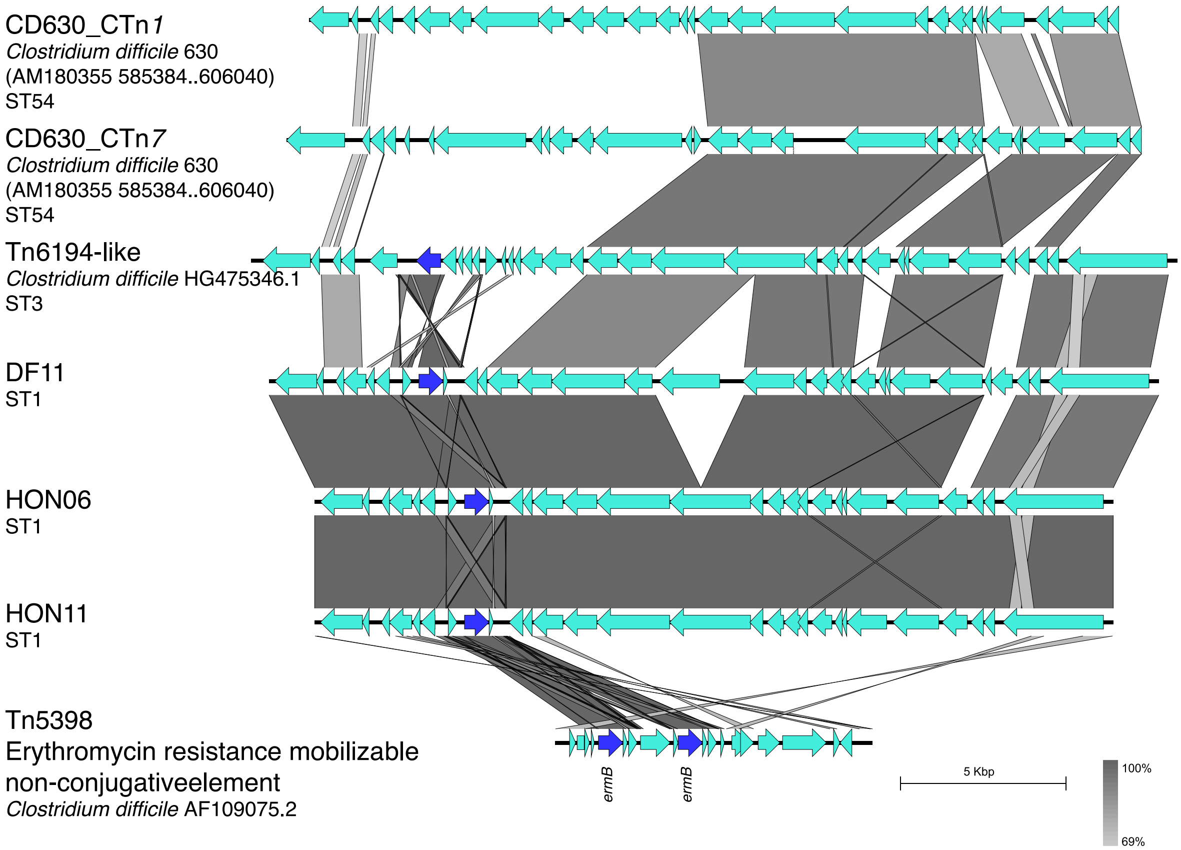

Supplementary Figure 11. Comparison of putative *ermB*⁺ MGEs identified in isolates DF11, HON06, and HON11.

A BLASTn search in the ICEBerg database revealed that the putative MGE with *ermB* (blue arrow) detected in DF11 and HON isolates is related to ICEs previously seen in other *C. difficile* strains. Accession numbers, strain names, and STs are shown at the left. A grayscale indicates the level of nucleotide sequence identity. InterProScan annotations related to MGEs are shown at the bottom of the annotated HON11 sequence.

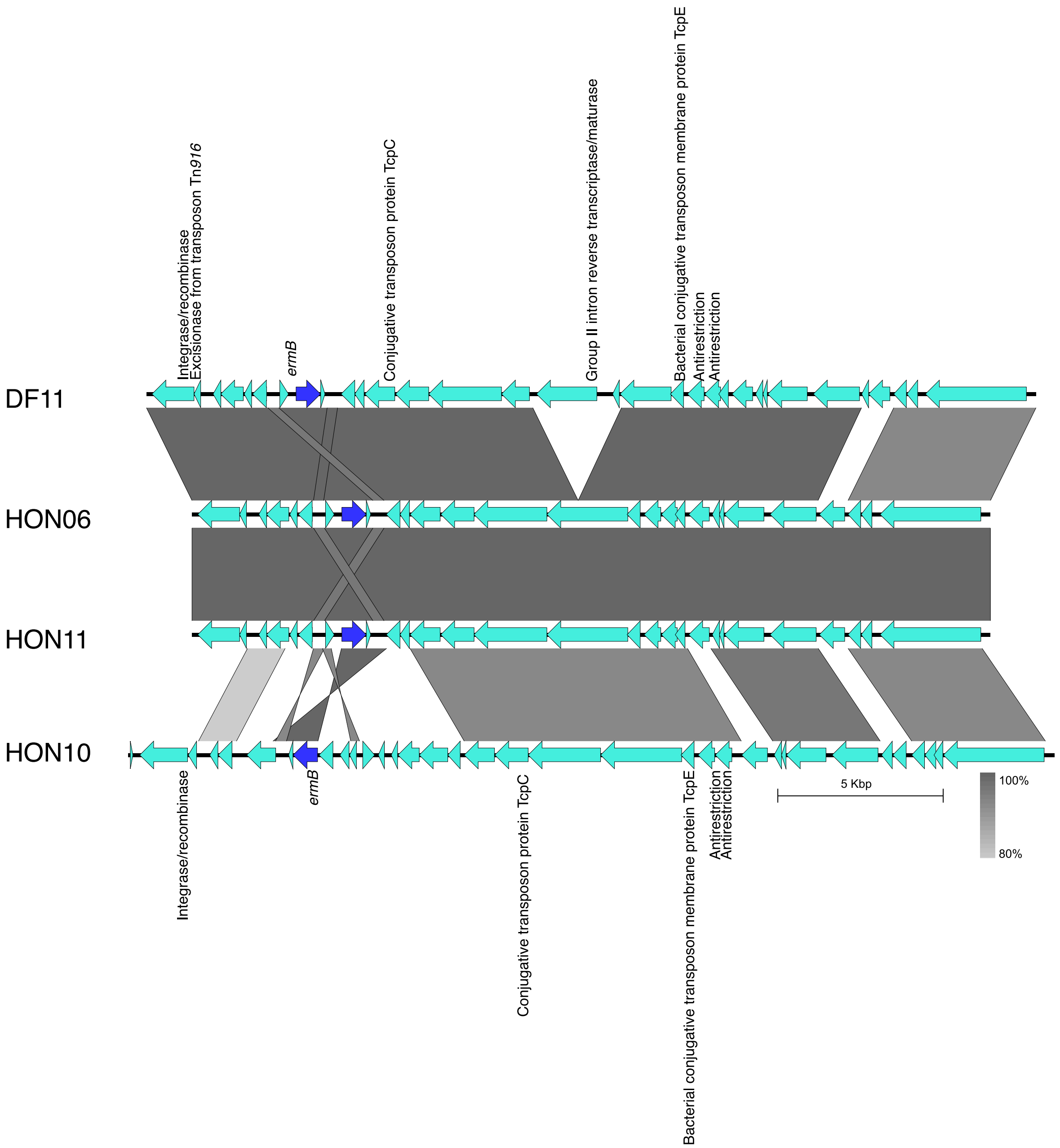

Supplementary Figure 12. Comparison of the putative *ermB*⁺ MGE acquired by isolates DF11, HON06, HON11 and HON10.

The *ermB*⁺ MGE of isolates DF11, HON06, HON11, and HON10 share features and are therefore related. A grayscale indicates the level of nucleotide sequence identity. InterProScan annotations related to MGEs are shown at the bottom of the annotated HON10 sequence.
